# Promoters of genes encoding β-amylase, albumin and globulin in food plants have weaker affinity for TATA-binding protein as compared to non-food plants: in silico analysis

**DOI:** 10.18699/VJGB-22-96

**Published:** 2022-12

**Authors:** O.V. Vishnevsky, I.V. Chadaeva, E.B. Sharypova, B.M. Khandaev, K.A. Zolotareva, A.V. Kazachek, P.M. Ponomarenko, N.L. Podkolodny, D.A. Rasskazov, A.G. Bogomolov, O.A. Podkolodnaya, L.K. Savinkova, E.V. Zemlyanskaya, M.P. Ponomarenko

**Affiliations:** Institute of Cytology and Genetics of the Siberian Branch of the Russian Academy of Sciences, Novosibirsk, Russi; Institute of Cytology and Genetics of the Siberian Branch of the Russian Academy of Sciences, Novosibirsk, Russia; Institute of Cytology and Genetics of the Siberian Branch of the Russian Academy of Sciences, Novosibirsk, Russia; Institute of Cytology and Genetics of the Siberian Branch of the Russian Academy of Sciences, Novosibirsk, Russia; Institute of Cytology and Genetics of the Siberian Branch of the Russian Academy of Sciences, Novosibirsk, Russia; Institute of Cytology and Genetics of the Siberian Branch of the Russian Academy of Sciences, Novosibirsk, Russia; Institute of Cytology and Genetics of the Siberian Branch of the Russian Academy of Sciences, Novosibirsk, Russia; Institute of Cytology and Genetics of the Siberian Branch of the Russian Academy of Sciences, Novosibirsk, Russia Institute of Computational Mathematics and Mathematical Geophysics of the Siberian Branch of the Russian Academy of Sciences, Novosibirsk, Russia; Institute of Cytology and Genetics of the Siberian Branch of the Russian Academy of Sciences, Novosibirsk, Russia; Institute of Cytology and Genetics of the Siberian Branch of the Russian Academy of Sciences, Novosibirsk, Russia; Institute of Cytology and Genetics of the Siberian Branch of the Russian Academy of Sciences, Novosibirsk, Russia; Institute of Cytology and Genetics of the Siberian Branch of the Russian Academy of Sciences, Novosibirsk, Russia; Institute of Cytology and Genetics of the Siberian Branch of the Russian Academy of Sciences, Novosibirsk, Russia; Institute of Cytology and Genetics of the Siberian Branch of the Russian Academy of Sciences, Novosibirsk, Russia

**Keywords:** food allergen, albumin, globulin, β-amylase, gene, promoter, common wheat Triticum aestivum L. (1753), plants, TATA-binding protein, TATA box, selection, in silico estimate, domestication, пищевые аллергены, альбумин, глобулин, β-амилаза, ген, промотор, мягкая пшеница Triticum aestivum L. (1753), растения, TATA-связывающий белок, TATA-бокс, доместикация, отбор, оценки in silico

## Abstract

It is generally accepted that during the domestication of food plants, selection was focused on their productivity, the ease of their technological processing into food, and resistance to pathogens and environmental stressors. Besides, the palatability of plant foods and their health benefits could also be subjected to selection by humans in the past. Nonetheless, it is unclear whether in antiquity, aside from positive selection for beneficial properties of plants, humans simultaneously selected against such detrimental properties as allergenicity. This topic is becoming increasingly relevant as the allergization of the population grows, being a major challenge for modern medicine. That is why intensive research by breeders is already underway for creating hypoallergenic forms of food plants. Accordingly, in this paper, albumin, globulin, and β-amylase of common wheat Triticum aestivum L. (1753) are analyzed, which have been identified earlier as targets for attacks by human class E immunoglobulins. At the genomic level, we wanted to find signs of past negative selection against the allergenicity of these three proteins (albumin, globulin, and β-amylase) during the domestication of ancestral forms of modern food plants. We focused the search on the TATA-binding protein (TBP)-binding site because it is located within a narrow region (between positions –70 and –20 relative to the corresponding transcription start sites), is the most conserved, necessary for primary transcription initiation, and is the best-studied regulatory genomic signal in eukaryotes. Our previous studies presented our publicly available Web service Plant_SNP_TATA_Z-tester, which makes it possible to estimate the equilibrium dissociation constant (KD) of TBP complexes with plant proximal promoters (as output data) using 90 bp of their DNA sequences (as input data). In this work, by means of this bioinformatics tool, 363 gene promoter DNA sequences representing 43 plant species were analyzed. It was found that compared with non-food plants, food plants are characterized by significantly weaker affinity of TBP for proximal promoters of their genes homologous to the genes of common-wheat globulin, albumin, and β-amylase (food allergens) (p < 0.01, Fisher’s Z-test). This evidence suggests that in the past humans carried out selective breeding to reduce the expression of food plant genes encoding these allergenic proteins.

## Abstract (ru)

Принято считать, что при доместикации пищевых растений отбор шел на урожайность, технологичность переработки в продукты питания, устойчивость к патогенам и стрессовым воздействиям окружающей среды. При этом также могли оцениваться вкусовые качества продуктов питания растительного происхождения и их ценность для здоровья. Однако неясно, проводил ли человек в прошлом наряду с положительным отбором на полезные свойства растений одновременно отбор против таких вредоносных свойств, как способность вызывать аллергические реакции. Этот вопрос становится все более актуальным по мере роста аллергизации населения как вызова современной медицине. В связи с этим селекционерами уже ведутся интенсивные исследования по созданию гипоаллергенных форм пищевых растений. В этой работе рассмотрены альбумин, глобулин и β-амилаза мягкой пшеницы Triticum aestivum L. (1753), идентифицированные ранее как мишени для атак иммуноглобулинов класса Е человека. Нашей целью было найти на геномном уровне следы отрицательного отбора в прошлом против гипераллергенности трех белков (альбумин, глобулин и β-амилаза) при одомашнивании предковых форм современных пищевых растений. Для этого мы сфокусировали поиск на сайте связывания ТАТА-связывающего белка (ТВР) как локализованном в узком районе [–70; –20] относительно старта транскрипции, консервативном, необходимом для первичной инициации транскрипции и наиболее изученном регуляторном сигнале в геномах эукариот. Ранее нами был создан свободно доступный веб-сервис Plant_SNP_TATA_Z-tester для оценки величин равновесной константы диссоциации (KD) комплексов ТВР с проксимальными промоторами генов растений по их последовательностям ДНК длиной 90 п. о. В настоящей работе с его помощью проанализированы 363 последовательности ДНК промоторов генов 43 видов растений. Обнаружено, что пищевые растения, в сравнении с непищевыми, характеризуются достоверно более низкой аффинностью ТBP к проксимальным промоторам их генов, гомологичных генам глобулина, альбумина и β-амилазы мягкой пшеницы как пищевых аллергенов ( p < 0.01, Z-критерий Фишера). Это свидетельствует об отборе при доместикации пищевых растений в прошлом на снижение уровня данных аллергенных белков.

## Introduction

Currently, the problem of food allergenicity is extremely relevant because the documented rapid growth of population allergization is becoming one of the key challenges for modern medicine (Prescott et al., 2022). In this regard, modern plant breeders are working in two directions: (1) creation of new hypoallergenic forms of agricultural food plants and (2) identification of new plant food allergens and of molecular mechanisms of their action (Hong et al., 2021; Cavazza et al., 2022).

The aim of our work was to search at the molecular genetic level for signs of negative selection against allergens during the domestication of ancestral forms of modern food plants. Three food allergens from common wheat Triticum aestivum L. (1753) were studied: β-amylase, albumin, and globulin, previously identified as targets of allergic reactions mediated by human class E immunoglobulins (Wang et al., 2021). The current study was conducted using our previously developed freely available Web service Plant_SNP_TATA_Ztester, which is designed to estimate the equilibrium dissociation constant (KD) of a complex of Arabidopsis thaliana (L.) Heynh. (1842) TBP-1 (hereafter: “plant TBP”) with a proximal promoter of various plant genes (Rasskazov et al., 2022). This tool was utilized to analyze 363 nucleotide sequences of proximal promoters of relevant genes from 43 plant species. As a result, compared to non-food plants, food plants were found to have significantly weaker affinity of plant TBP toward promoters of genes homologous to common-wheat genes of β-amylase, albumin, and globulin (food allergens). These data indicate that in the past, selection was carried out by humans for reducing the expression of food plant genes encoding allergenic proteins when such plants were domesticated.

## Materials and methods

Nucleotide sequences of plant gene promoters analyzed in this work. Three allergenic proteins from common wheat T. aestivum were investigated: β-amylase, albumin, and globulin, which have previously been experimentally identified as targets for human class E immunoglobulins (Wang et al., 2021). From the GenBank database (Benson et al., 2015), nucleotide sequences of 90 bp proximal promoters were retrieved that are located immediately upstream of transcription start sites of plant genes homologous to the genes of β-amylase, albumin, and globulin from common wheat T. aestivum. After the exclusion of promoter DNA sequences with unknown nucleotides w, s, r, y, k, m, b, d, h, v, and n (according to the nomenclature of (IUPAC-IUB…, 1970)), we had 363 promoter sequences belonging to 43 plant species. Then, all 43 plant species were categorized into two nonoverlapping groups: group I, represented by 235 proximal promoters from 28 food plant species for which there was information about their centuries-old use by humans as foods (Table 1), and group II, represented by 128 proximal promoters from non-food plants (the other 15 species) (Table 2).

**Table 1.**
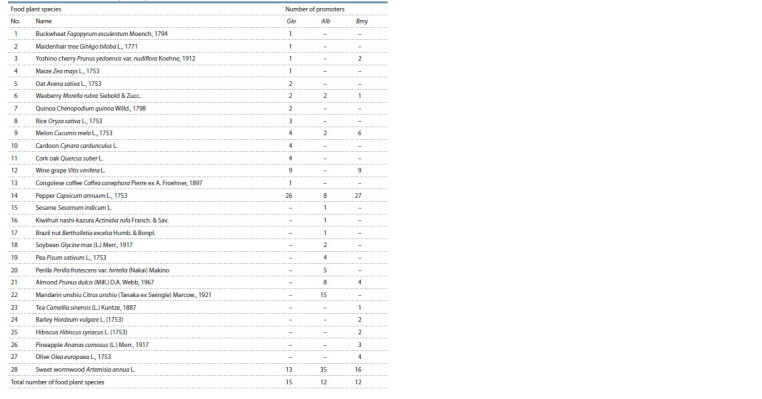
Characteristics of 235 nucleotide sequences of proximal promoters of food plant genes homologous to the studied globulin (Glo), albumin (Alb), and β-amylase (Bmy) genes from common wheat T. aestivum

**Table 2.**
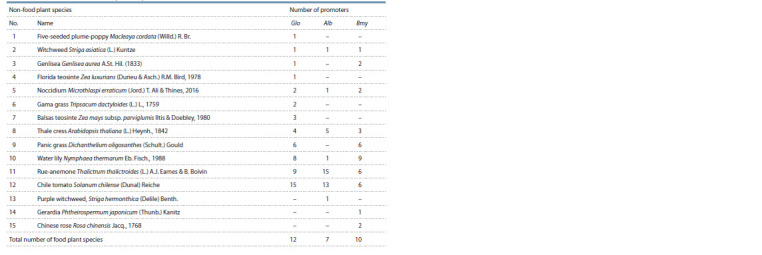
Characteristics of 128 nucleotide sequences of proximal promoters from non-food plant genes homologous to the studied globulin (Glo), albumin (Alb), and β-amylase (Bmy) genes from common wheat T. aestivum

Nucleotide sequence analysis of proximal promoters of plants. Using Web service Plant_SNP_TATA_Z-tester (Rasskazov et al., 2022), which we have created earlier, we calculated KD (in moles per liter; M) for complexes of plant TBP with each promoter by means of the nucleotide sequence of each promoter (characterized in Tables 1 and 2).

The calculations were performed in accordance with our previously formulated model of three-step binding of TBP to a promoter (i) TBP slides along the double helix of promoter DNA (Coleman, Pugh, 1995) ↔ (ii) TBP stops at a potential site of TBP binding (Berg, von Hippel, 1987; Bucher 1990) ↔ (iii) the TBP/promoter complex is stabilized by bending of the DNA double-helix axis at a right angle (Flatters, Lavery, 1998), as subsequently demonstrated experimentally in vitro (Delgadillo et al., 2009).

Statistical analysis. In this work, using standard software package Statistica (Statsoft™, USA), we averaged the Plant_SNP_TATA_Z-tester-generated (Rasskazov et al., 2022) estimates of KD – for complexes of plant TBP with promoters of β-amylase, albumin, and globulin genes – for food and nonfood plants separately. On the basis of these data, statistical significance of differences between food and non-food plants was evaluated by Fisher’s Z-test.

## Results

Globulin

Table 3 presents the in silico estimates of KD for complexes of plant TBP with 74 proximal promoters of globulin genes from 15 food plant species in comparison with 53 such promoters from 12 non-food plant species, as determined using Plant_SNP_TATA_Z-tester (Rasskazov et al., 2022). One can see in this table that in the case of food plants, the estimates of KD for complexes of plant TBP with promoters of these genes varied from 1.67 ± 0.12 (mean ± SEM) to 6.75 ± 5.23 nM, with an average of 2.97 ± 0.21 nM, whereas for non-food plants, these values varied from 1.25 ± 0.06 to 3.33 ± 0.23 nM, with an average of 2.15 ± 0.08 nM.

**Table 3.**
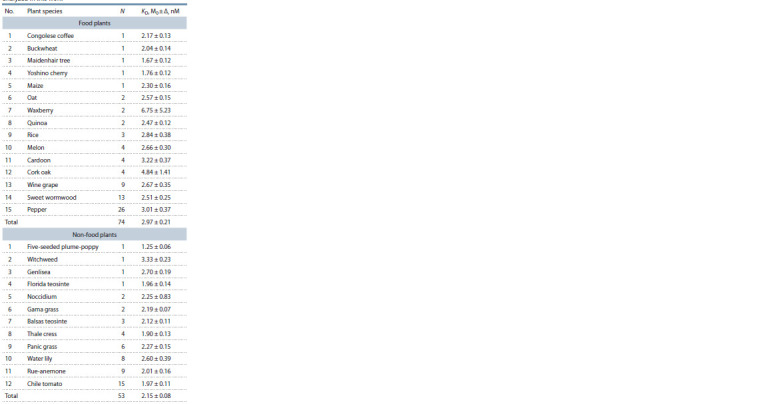
Arithmetic mean estimates (M0) of the equilibrium dissociation constant (KD) of complexes between plant TBP and 90 bp proximal promoters of the plant globulin genes analyzed in this work Notе. Here and in Tables 4 and 5: N – total number of the promoter studied; M0 – arithmetic mean score; Δ – standard error of the mean (SEM).

**Fig. 1.**
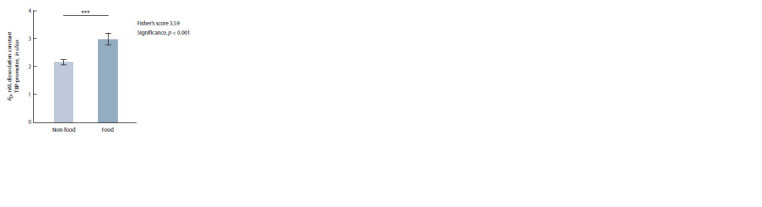
The statistically significant difference between the studied food plants and non-food plants in the in silico estimates of KD for complexes of plant TBP with 90 bp proximal promoters of their genes encoding globulins. Here and in Fig. 2: *** statistical significance p < 0.001 according to Fisher’s Z-test.

In Fig. 1, arithmetic mean estimates of KD for complexes of plant TBP with globulin-coding gene promoters are compared between two groups (food and non-food plants) by Fisher’s Z-test. The difference between the groups was significant, with Z = 3.59 and p < 0.001.

Albumin

Table 4 shows data obtained by Web service Plant_SNP_ TATA_Z-tester (Rasskazov et al., 2022) regarding estimates of KD for complexes of plant TBP with 84 albumin gene promoters from 12 food plant species and with 37 promoters from 7 non-food plant species. As readers can see in this table, in the case of food plants, the estimates of KD of TBP-promoter complexes for these genes ranged between 1.65 ± 0.12 and 4.49 ± 1.39 nM (average: 3.10 ± 0.22 nM), whereas for nonfood plants, they ranged from 1.65 ± 0.05 to 2.70 ± 0.22 nM (average: 2.18 ± 0.10 nM).

**Table 4.**
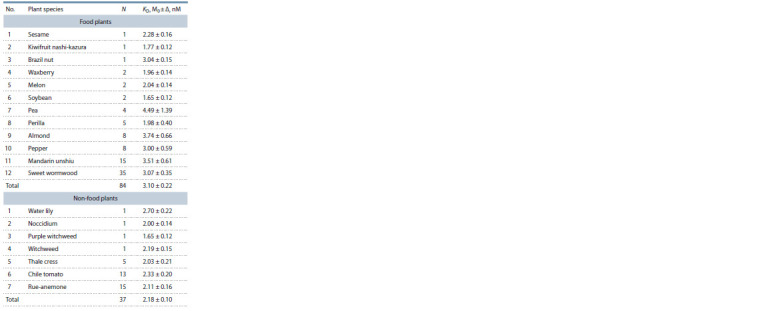
Arithmetic mean estimates (M0) of the equilibrium dissociation constant (KD) for complexes between plant TBP and 90 bp proximal promoters of the plant albumin genes investigated in this work

**Fig. 2.**
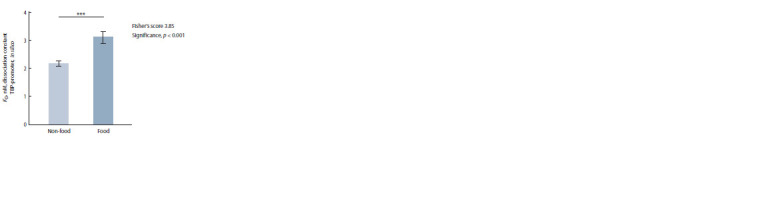
The statistically significant difference between the studied food plants and non-food plants in the in silico estimates of KD for the complexes of plant TBP with 90 bp proximal promoters of their genes encoding albumins.

A comparison of the two groups (food and non-food plants) by Fisher’s Z-test is displayed in Fig. 2. Here one can see a significant difference between food plants and non-food plants (Z = 3.85, p < 0.001).

β-Amylase

**Fig. 3.**
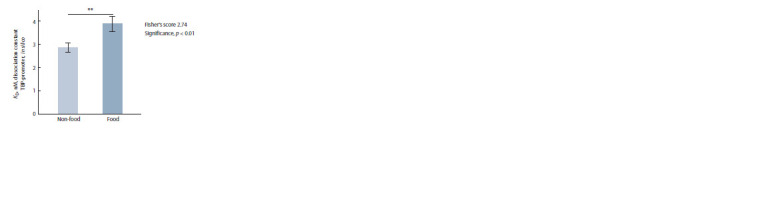
The statistically significant difference between the studied food plants and non-food plants in the in silico estimates of KD for the complexes of plant TBP with 90 bp proximal promoters of their genes encoding β-amylases. ** Statistical significance p < 0.01 according to Fisher’s Z-test.

Table 5 lists estimated KD values of complexes of plant TBP with 77 proximal promoters of β-amylase genes from 12 food plant species and with 38 promoters from 10 non-food plant species, as calculated by Web service Plant_SNP_TATA_ Z-tester (Rasskazov et al., 2022). For food plants, this table presents the range of KD from 1.30 ± 0.09 to 8.77 ± 7.36 nM, with an arithmetic mean of 2.85 ± 0.21 nM, whereas for nonfood plants, the range of KD was found to be 1.66 ± 0.32 to 6.75 ± 5.23 nM, with an average of 3.89 ± 0.32 nM. Fig. 3 presents a comparison between the analyzed food and nonfood plants by Fisher’s Z-test, according to which these groups are statistically significantly different at Z = 2.74 and p < 0.01.

**Table 5.**
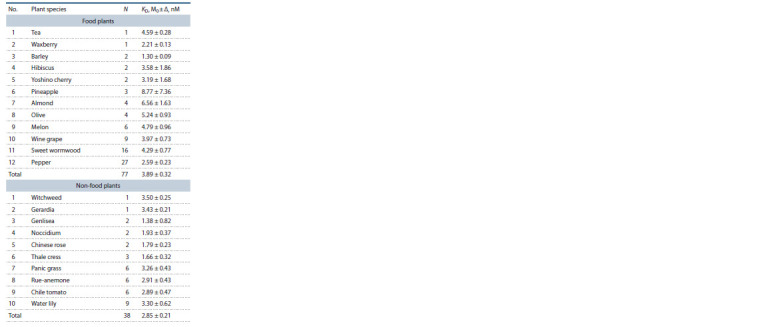
Arithmetic mean estimates (M0) of the equilibrium dissociation constant (KD) of complexes between plant TBP and 90 bp proximal promoters of the plant β-amylase genes examined in this work

## Discussion

It is well known that in the process of spontaneous domestication of ancestral forms of modern food plants, the selection was primarily based on their economically valuable traits, such as productivity, resistance to pathogens and to environmental stressors, and the ease of technological processing into final food products. Additionally, during the plant domestication, humans assessed the palatability of food products and their benefits for health

It remains unclear whether in addition to the positive selection for the beneficial properties of agricultural plants, there was also simultaneous selection against their detrimental properties, which include allergenicity of the dishes prepared from these plants. To answer this question, we concentrated on the search for molecular genetic selection markers related to structural and functional organization of proximal promoters of plant genes.

Accordingly, plant genes were analyzed that are homologous to three T. aestivum genes encoding food allergens β-amylase, albumin, and globulin, earlier identified as targets for human IgE (Wang et al., 2021). Thus, 363 homologous genes were investigated belonging to 28 and 15 species of food plants and non-food plants, respectively. With the help of Web service Plant_SNP_TATA_Z-tester (Rasskazov et al., 2022), for each homologous gene, KD of the complex of plant TBP with this gene’s proximal promoter was computed.

Interest in the TBP protein and its binding site in the proximal promoter (canonical form: the TATA box) is due to the fact that they play a key role in the initiation of eukaryotic gene transcription. It has been experimentally established (Coleman, Pugh, 1995) that TBP slides along the DNA double helix owing to nonspecific affinity between them: KD ~ 10–5 M (Hahn et al., 1989). TBP then stops at a site of TBP binding because of their mutual molecular recognition (Berg, von Hippel, 1987; Bucher, 1990) mediated by stronger (specific) affinity of TBP for this site: KD ~ 10–9 M (Hahn et al., 1989). Next, under the action of TBP, the DNA double helix melts at the site of TBP binding, and kinking of the DNA axis at a right angle takes place, which stabilizes the TBP-promoter complex (Flatters, Lavery, 1998). The resultant TBP-promoter complex is considered an obligatory DNA anchor, which is required for the binding of RNA polymerase II (Muller et al., 2001; Martianov et al., 2002; Choukrallah et al., 2012; Rhee, Pugh, 2012) as a key step in the assembly of the transcription preinitiation complex (Auble, 2009) responsible for basal transcription (Fire et al., 1984). Due to the key importance of TATA boxes, mutations located in proximal promoters have a well-pronounced effect on the magnitude of gene expression (Savinkova et al., 2009).

The molecular mechanism underlying the binding of TBP to a promoter of various eukaryotic genes via the three successive steps was first proposed by P. Ponomarenko et al. (2008) and later confirmed experimentally (Delgadillo et al., 2009). Based on this mechanism, a bioinformatic model was devised previously for calculating a change in KD (of a complex between TBP and a proximal promoter of a eukaryotic gene) for a polymorphism of the TBP-binding site(s) in the promoter as compared to the wild type (Ponomarenko et al., 2009). Results of computations based on this model have been confirmed by independent ex vivo experiments on cell cultures transfected with the pGL4.10 plasmid (Promega, USA) carrying a wild-type or mutant promoter inserted before a luciferase reporter gene (Ponomarenko et al., 2017) as well as in vitro in real time (Arkova et al., 2017) by means of stopped-flow spectrometer SX.20 (Applied Photophysics, UK) under equilibrium conditions (Savinkova et al., 2013) and under nonequilibrium conditions (Drachkova et al., 2014) with the help of an electrophoretic mobility shift assay. As a result of such comprehensive verification of this bioinformatic model, on its basis, the Web service Plant_SNP_TATA_Z-tester (Rasskazov et al., 2022) was created, which was employed in the current project for estimating KD of complexes of plant TBP with proximal promoters of genes from food and non-food plants.

Our analysis revealed that in comparison with non-food plants, food plants are characterized by significantly weaker affinity of TBP for promoters of genes homologous to common-wheat β-amylase, albumin, and globulin (food allergens) ( p < 0.01, as estimated by the above software and Fisher’s Z-test). When interpreting the obtained results, let us take into account the experimentally proven fact that the level of expression of eukaryotic genes increases with enhancement of the affinity of TBP for the promoters of these genes (Mogno et al., 2010). This observation allows us to interpret the food plants’ weaker TBP affinity – for promoters of genes homologous to genes of food allergens (commonwheat β-amylase, albumin, and globulin) in comparison with non-food plants – as evidence of selection by humans for low amounts of these allergenic proteins in food plants in the past, during the domestication of the plants.

## Conclusion

In this work, DNA sequences of proximal promoters of genes homologous to genes of food allergens (Wang et al., 2021) were consistently analyzed in silico for the first time for food compared to non-food plants. As a result, weaker in silico affinity of TBP was observed for promoters of the investigated food plant genes as compared to genes of non-food plants. This finding is suggestive of artificial selection – in antiquity, for the purpose of reducing the expression of food plant genes encoding allergenic proteins – carried out by humans in the course of domestication of plants as food products.

## Conflict of interest

The authors declare no conflict of interest.
